# The Relationship between Therapeutic Alliance and Quality of Care in Patients with Advanced Cancer in Spain

**DOI:** 10.3390/curroncol30040273

**Published:** 2023-03-24

**Authors:** Verónica Velasco-Durántez, Luka Mihic-Góngora, Sara Coca-Membribes, Rocío Galán-Moral, Ana Fernández-Montes, Oscar A. Castillo-Trujillo, Elena Sorribes, Alicia Quilez, Laura Puntí-Brun, Paula Jiménez-Fonseca, Caterina Calderon

**Affiliations:** 1 Department of Medical Oncology, Hospital Universitario Central de Asturias, ISPA, 33011 Oviedo, Spain; 2 Department of Medical Oncology, Hospital Universitario de Canarias, 38320 Tenerife, Spain; 3 Department of Medical Oncology, Hospital General Universitario de Ciudad Real, 13005 Madrid, Spain; 4 Department of Medical Oncology, Complejo Hospitalario Universitario de Ourense, 32005 Ourense, Spain; 5 Faculty of Psychology, University of Barcelona, 08007 Barcelona, Spain; 6 Department of Medical Oncology, Hospital Can Misses, 07800 Eivissa, Spain; 7 Department of Medical Oncology, Consorci Sanitari del Maresme, 08304 Mataró, Spain

**Keywords:** advanced cancer, alliance therapeutic, prognosis, satisfaction, communication

## Abstract

The therapeutic alliance is an important factor in successful cancer treatment, particularly for those with advanced cancer. This study aims to determine how the therapeutic alliance relates to prognostic preferences and satisfaction with the physician and medical care among patients with advanced cancer. We conducted a cross-sectional study to explore the therapeutic relationship, trust, satisfaction with healthcare, and prognostic preferences among 946 patients with advanced cancer at 15 tertiary hospitals in Spain. Participants completed questionnaires with self-reported measures. Most were male, aged > 65 years, with bronchopulmonary (29%) or colorectal (16%) tumors and metastatic disease at diagnosis. Results revealed that 84% of patients had a good therapeutic alliance. Collaborative and affective bond was positively associated with a preference to know the prognosis and satisfaction with care and decision. There was no difference in a therapeutic alliance based on clinical or sociodemographic factors. The therapeutic alliance between patient and physician is essential for successful treatment outcomes and better overall satisfaction. Therefore, it is vital for healthcare providers to focus on establishing and maintaining a strong relationship with their patients. To achieve this, transparency and care should be prioritized, as well as respect for the preferences of patients regarding the prognosis of their illness.

## 1. Introduction

Due to recent advances in cancer treatments, the course of the disease and prognosis for cancer patients have changed, allowing for a relatively long period of life for those with advanced cancer [1]. Unfortunately, long-term experience with cancer and its treatments can lead to physical, psychological, emotional, and practical problems [2,3,4]. Additionally, tumor stage can affect the types of symptoms and experiences patients have during treatment; those with unresectable advanced cancer often have different experiences than those with resected localized cancer [5]. This creates a difficult situation for both the patient and the attending physicians [5,6].

The doctor–patient therapeutic alliance is a key element of personalized and quality care for patients with advanced cancer [7,8]. This alliance is characterized by shared goals, mutual understanding, care, trust, respect, and recognition of the patient’s personality, as well as honesty and competence from the doctor [1,9,10]. Developing a good therapeutic relationship is an essential skill for doctors to improve the quality of their services, and this contributes to more successful outcomes. For example, a strong doctor–patient relationship is necessary for informed decision-making [11], increasing patient satisfaction, empowerment [12,13], and adherence to treatment [14].

Research has demonstrated that a strong relationship between a doctor and a patient can have a beneficial effect on treatment outcomes, the alleviation of symptoms, patient health, the patient’s satisfaction with their care, and a reduction in litigation cases associated with inadequate medical practices [9,15,16]. Studies have found that a positive connection between the patient and their oncologist is linked with better adherence to treatment and a decreased risk of suicidal ideation in the patient [17], which is also predictive of higher quality of life and better adaptation to the illness [6,9,18]. A meta-analysis of physician communication and treatment adherence revealed that patients whose doctors had poor communication strategies had a 19% higher risk of non-compliance than those whose doctors communicated effectively [14]. Other factors have also been used to assess the quality of the doctor–patient relationship, such as trust in the oncologist or satisfaction with the care they received [12,13].

The present study seeks to examine the relationship between therapeutic alliance and the patient’s desire to know the prognosis, satisfaction with the physician and medical care received, and trust in the physician in advanced cancer patients. We hypothesize that a stronger therapeutic alliance will be associated with greater satisfaction with the information given and treatment decision-making, as well as an increased level of trust in the physician and a desire to know the prognosis on the part of the patient. This research is significant as the evaluation of the physician–patient therapeutic relationship is becoming increasingly important for assessing the quality and satisfaction of care received [9,15,16]. However, there is currently a lack of studies on physician–patient alliance and satisfaction with communication with the physician, as well as treatment decision-making in advanced cancer patients. The aim of the present study, therefore, is to analyze the patient’s prognostic preferences, satisfaction with the physician and medical care received and its relationship with the therapeutic alliance in advanced cancer patients.

## 2. Materials and Methods

### 2.1. Study Design and Population

A cross-sectional study was conducted at 15 medical oncology departments in different hospitals distributed throughout Spain between February 2020 and October 2022 (Table A1, see Appendix A section). The consecutive sample consisted of advanced cancer patients who were recruited during their first appointment with the medical oncologist. Inclusion criteria were individuals who were at least 18 years of age with histologically confirmed advanced cancer and were ineligible for curative treatment options. Patients were excluded if they had a physical condition, comorbidities, and/or age that contraindicated antineoplastic treatment in the attending oncologist’s opinion; those who had received cancer treatment for another advanced cancer in the previous two years or with any underlying personal, family, sociological, geographical, and/or medical condition that could hinder their ability to participate in the study, i.e., those with cognitive impairment or severe deterioration of general status due to cancer or other causes that prevent them from understanding and reasoning what is asked in the questionnaires. All participants provided informed consent before being included in the study. The study was approved by the Ethics Review Committees at each institution and by the Spanish Agency of Medicines and Health Products (AEMPS; identification code: ES14042015). Data were collected through the completion of questionnaires, and clinical data were obtained from medical records. The procedures for collecting data were similar at all the hospitals, and the data regarding the participants were obtained from the centers where they received treatment. Participation was voluntary, anonymous, and did not affect patient care. Data were collected and updated by the medical oncologist through a platform (www.neoetic.es, accessed on 9 March 2023).

### 2.2. Description of Variables

The Bioethical Section of the Spanish Society of Medical Oncology (SEOM) conducted a multicenter, cross-sectional study. Patients with recently diagnosed cancer that cannot be surgically removed and who were eligible for systemic anticancer treatment according to their oncologist’s judgment were included in the study. Patients filled out questionnaires to assess their sociodemographic status, therapeutic alliance, trust in their oncologist, satisfaction with healthcare, and prognostic preferences using a standardized self-report form. Cancer and treatment information was obtained by the oncologist, by interviewing the patient and by reviewing their medical records. The oncologist classified the patient’s functional status according to the Eastern Cooperative Oncology Group (ECOG) scale, which ranges from 0 (asymptomatic) to 5 (deceased). Any value was accepted as long as the oncologist thought the patient was eligible for systemic treatment. The oncologist gave the questionnaire to the participant during their consultation and explained the systemic antineoplastic treatment. The patient filled out the questionnaire at home before starting the treatment.

The therapeutic alliance was measured using the Scale to Assess the Therapeutic Relationship-Patients version (STAR-P) [19]. This self-rating scale consists of 12 questions divided into two subscales: collaborative bond and affective bond [20]. The collaborative bond subscale comprises six items. It measures shared understanding of goals and mutual openness, while the affective bond subscale measures the patient’s perception of the clinician, such as being impatient, authoritarian, or untruthful. The affective bond questions also comprise six items, and three of them were reverse-scored. Each item is rated on a five-point Likert scale ranging from 0 (strongly disagree) to 4 (strongly agree). A total score was calculated by adding the subscales together. The total scores range from 0 to 48, and higher scores indicated a better therapeutic alliance. The internal consistency of the overall score was Cronbach’s alpha = 0.85 in the sample.

Previously, nine items derived from the Consumer Assessment of Healthcare Providers and Systems (CAHPS) were used to measure patients’ satisfaction with healthcare [21,22]. Six of these items related to physician communication (e.g., listening carefully, explaining things in an understandable way, providing information about treatments, encouraging questions, and showing courtesy and respect) and three related to satisfaction with decision-making (e.g., satisfaction with information, satisfaction with how decisions were made, and satisfaction with the decisions made). Each item was rated on a five-point Likert scale, with higher scores indicating better communication with physicians and satisfaction with decision-making [23]. The internal consistency of the overall scores was Cronbach’s α = 0.86 in our sample.

The Trust in Oncologist Scale (TiOS-SF) five-item short version was used to measure whether the oncologist was seen as trustworthy [12]. The scale consists of five statements that patients are asked to rate on a five-point Likert scale, ranging from “strongly- disagree” to “strongly agree”. The scores for each item are summed up to create a total score, with higher scores indicating a greater level of trust in the oncologist. A score below 19 has been suggested as indicative of lower trust in the oncologist [12]. The TiOS overall had high reliability (α = 0.92) [12]. 

The Patient’s Prognostic Preferences (PPP) is a scale used to assess the level of information that cancer patients want their oncologists to share with them about their illness. The scale consists of five items, which are rated on a five-point Likert scale ranging from “strongly disagree” “to strongly agree”. The scale measures the level of detail that patients want their oncologist to provide about their diagnosis, prognosis, and treatment options. A higher score reflects a greater desire for detailed information. The internal consistency of the overall PPP score in this study was Cronbach’s α = 0.82.

### 2.3. Statistical Methods

Descriptive statistics were obtained for clinical and sociodemographic variables. Independent sample t-tests and analyses of variance (ANOVA) were used to compare the therapeutic alliance score across the study population’s demographic and clinical characteristics. The Pearson correlation was used to assess the association between the therapeutic alliance and quality of care (satisfaction with the received information, satisfaction with decisions made, trust in the oncologist, and patient’s preferences for knowing the prognosis). The association between therapeutic alliance and quality of care was further examined through regression analysis, with therapeutic alliance used as the independent variable and age, sex, and tumor site used as covariates. Statistical significance was set at *p* < 0.05, and all statistical analyses were performed using SPSS version 26 (IBM Corp., Armonk, NY, USA).

## 3. Results

### 3.1. Relationship between Therapeutic Alliance and Clinical/Sociodemographic Characteristics 

A total of 946 participants were included in the study, recruited by 30 medical oncologists. Some 78% of these specialists were female, and the mean age was 38 ± 8.5 years, with 13.8 ± 8.5 years of experience in caring for cancer patients. Most were super-specialists (69%) working at a public teaching hospital. Most patients were male, 68% were married, 46% had completed junior high school, and 51% were retired or unemployed. The mean age of the patients was 60.1 years (standard deviation [SD]: 10.1). The most common cancers were bronchopulmonary (29%), colorectal (16%), and pancreas (9%), with adenocarcinoma histology being the most prevalent (64%). Most cancers were diagnosed in stage IV (80%), and the most frequent treatment was chemotherapy alone or combined with other therapeutic modalities (89%). The analysis revealed that approximately 45% of the sample had an estimated survival of fewer than 18 months. Additionally, no significant differences were identified among diverse clinical and sociodemographic groups with respect to the therapeutic alliance, as shown in Table 1. Of the advanced cancer patients, 84% (*n* = 790) believed they had a good therapeutic alliance with their doctor (STAR, PD > 31), and 92% of the patients trusted their oncologist (TIOS, PD > 20).

### 3.2. Associations between Therapeutic Alliance and Psychological Variables

The therapeutic alliance was positively associated with its two sub-scales, particularly with the collaborative bond (M = 20.0 SD = 4.4) (*r* = 0.897, *p* < 0.001), more so than with the affective bond (M = 19.0, SD = 3.7) (*r* = 0.854, *p* < 0.001). Additionally, the therapeutic alliance was found to be associated with satisfaction with decision-making and physician communication, trust in the oncologist, and preference to know the prognosis. It was found that collaborative and affective bonds had a positive association with the preference to know prognosis, according to Table 2.

Univariate analysis revealed that patient satisfaction with decision-making and physician communication, preference to know the prognosis and trust in the oncologist were favorable predictors of the better therapeutic alliance (*F* = 69.631, *p* = 0.001, adjusted R^2^ =0.342). This significance was maintained after adjusting for covariates such as age, sex, and tumor site, as seen in Table 3.

## 4. Discussion

This study provides information on the therapeutic alliance and satisfaction with care among cancer patients and their oncologists. Out of the patients with advanced cancer, 84% reported having a good therapeutic alliance and being satisfied with their oncologists’ visits, which is congruent with findings in other medical care fields [24,25]. Some studies suggest that medical service patients may not differentiate between their satisfaction with received care and its socio-emotional aspects [26,27]. Our findings indicate that the various aspects of the therapeutic alliance are closely related, particularly those which reflect positive aspects of the doctor–patient relationship, such as collaboration and shared understanding of therapeutic goals, and emotional aspects, particularly in cases where these aspects reflect subjective issues in the doctor–patient relationship, such as the patient’s perception of their doctor being impatient, authoritarian, or not sharing all necessary information.

Our findings suggest that the therapeutic alliance is closely linked to a patient’s confidence and satisfaction in the doctor’s provided information and treatment decision-making, as well as in their preference for knowing their prognosis. We found that the more satisfied cancer patients were with the decision-making and information received, the greater their desire to know their prognosis and their confidence in the doctor, all of which contributed to a better therapeutic alliance with the doctor. The variable that had the most significant effect on improving the therapeutic alliance was satisfaction with decisions, while the least was the patient’s preference to know their prognosis. The patient’s preference to know their prognosis was positively associated with both the collaborative bond with the doctor, reflecting a shared understanding of goals and an open mutual experience, as well as the affective bond, which would reflect the patient’s perception that their doctor shares all information with them. This is consistent with prior research suggesting that a good relationship with the doctor is linked to service satisfaction and adherence to treatment [14,16,20,28], while a mismatch between the patient’s desire for involvement in decision-making and a lack of opportunity to do so can lead to a lower therapeutic alliance and dissatisfaction with care received [28]. Patient satisfaction may be closely linked to their desire to be involved in decision-making regarding their treatment [9,29,30,31,32]. Consequently, it is essential for healthcare providers to acknowledge that enabling the patient to participate in their treatment decisions can have a positive effect on the patient’s satisfaction with the care they receive, as well as permit the establishment of a solid therapeutic relationship.

Our study did not find any major differences in the therapeutic alliance depending on sociodemographic or clinical variables. This finding is consistent with the results of other studies that have not indicated a relationship between these variables and the therapeutic alliance [33]. Nevertheless, some studies have suggested that the therapeutic alliance may be enhanced by age [34], that married individuals may have a better therapeutic alliance than single patients [30], and that males may have a better therapeutic alliance than females [34]. These results suggest that further research should be conducted to investigate whether there are sociodemographic predictors that are associated with an improved therapeutic alliance and patient satisfaction with medical care.

There are some **limitations** other than the selection bias already mentioned. First, the cross-sectional design limited the ability to draw inferences with respect to the causal direction of associations between therapeutic alliance and satisfaction with the care received, which were considered to be dynamic factors. Second, it is possible that the participating oncologists were biased due to their interest in communication issues. This interest may influence their interaction with patients, which could result in a patient response bias. Third, we have not considered some factors from the oncologist’s perspective that may favor the development of the therapeutic relationship, such as being curious, optimistic, patient, having a sense of humor, age, and sex, among others. Fourth, the analysis of the therapeutic alliance between patients and their oncologists does not permit us to generalize the findings to other healthcare professionals. Nevertheless, to the best of our knowledge, this study is one of the few studies that have examined the effect of satisfaction with the care received on therapeutic alliance. Additionally, this study reflected the routine outpatient clinical setting and considered confounding factors that could affect therapeutic alliance.

## 5. Conclusions

In conclusion, the therapeutic alliance between patients with advanced cancer undergoing active oncological treatment was affected by the satisfaction of the patient with the physician (the information provided and the satisfaction with decisions taken), as well as the trust in the physician and the patient’s preference to know their prognosis. These findings suggest that advanced cancer patients need more than collaboration and positive contributions from their physicians; it would involve understanding and respect for the patient’s desires to take part in the decision-making process regarding their treatment, which could also improve the therapeutic alliance. Future studies could examine whether involving patients in the decision-making process with their oncologists improves satisfaction and leads to better commitment and adherence to treatment recommendations.

## Figures and Tables

**Table 1:** Baseline characteristics and alliance therapeutic score (*n* = 946).

Variables		*n* (%)	STAR Score	Statistics	
				t/f	*p*
**Sex**	Male	519 (55)	38.6 (7.1)	3.276	0.071
	Female	427 (45)	39.5 (7.1)		
**Age (years)**	≤65.0	430 (46)	38.9 (7.0)	0.001	0.978
	≥65.1	516 (54)	39.0 (7.2)		
**Marital**	Married or partnered	644 (68)	39.0 (7.2)	0.098	0.754
	No partnered	302 (32)	38.9 (7.0)		
**Educational level**	Primary school or less	430 (46)	39.1 (7.1)	0.002	0.963
	High school or greater	516 (54)	39.0 (7.4)		
**Employ**	Yes	468 (49)	39.2 (7.2)	0.523	0.470
	No	478 (51)	38.8 (7.0)		
**Tumor site**	Bronchopulmonary	278 (29)	39.0 (7.2)	0.027	0.994
	Colorectal	153(16)	38.8 (6.7)		
	Pancreas	88 (9)	39.0 (6.9)		
	Others	427 (46)	39.0 (7.1)		
**Histology**	Adenocarcinoma	610 (64)	39.2 (7.3)	0.907	0.341
	Others	336 (36)	38.7 (6.8)		
**Stage**	Locally advanced	188 (20)	39.6 (7.2)	1.774	0.183
	IV	758 (80)	38.8 (7.1)		
**Estimated survival (months)**	<18	424 (45)	38.7 (7.1)	1.154	0.283
	≥18	521 (55)	39.2 (7.1)		
**Systemic treatment**	Chemotherapy (CT)	499 (53)	38.9 (6.9)	0.329	0.858
	Targeted therapy	61(6)	38.0 (6.9)		
	Immunotherapy	50 (5)	39.5 (6.9)		
	Others	336 (36)	36.8 (6.3)		
**Elixhauser comorb. index**	≤4	382 (40)	39.1 (7.2)	0.221	0.638
	>4	564 (60)	38.9 (7.1)		
**ECOG**	0–1	202 (62)	39.4 (7.3)	2.270	0.132
	2–3	355 (38)	38.7 (6.9)		

Bold values indicate significance at the 5% level. Abbreviations: Eastern Cooperative Oncology Group (ECOG).

**Table 2:** Correlations between therapeutic alliance and psychological variables (*n* = 946).

		Therapeutic Alliance (STAR-P)		
Variable	M ± SD	Total	Collaborative Bond	Affective Bond
STAR-P. Total therapeutic alliance	39.0 ± 7.1	--	0.897 **	0.854 **
CAHPS. Satisfaction with decision-making	10.6 ± 2.1	0.517 **	0.544 **	0.347 **
CAHPS. Satisfaction with physician communication	19.1 ± 2.3	0.496 **	0.517 **	0.338 **
TiOS. Trust in the physician	24.0 ± 2.5	0.462 **	0.471 **	0.328 **
PPP. Preference to know the prognosis	17.4 ± 3.3	0.253 **	0.299 **	0.131 **

Abbreviations: STAR-P, Scale to assess the Therapeutic Relationship-Patients version; CAHPS, Satisfaction with decision-making and physician communication; PPD, Patient’s Prognostic Preferences; TiOS, Trust in the oncologist scale. M, Mean. SD, Standard Deviation. Table entries are Pearson correlation coefficients; significant at ** *p* < 0.01.

**Table 3:** Linear regression models for therapeutic alliance predictors (*n* = 946).

	Therapeutic Alliance (STAR-P)				
Predictors	B	*Beta*	*t*	*p*	*R*^2^ Adjusted
CAHPS. Satisfaction with decision-making	0.973	0.289	8.077	0.001	0.342
CAHPS. Satisfaction with communication	0.544	0.177	4.624	0.001	
PPP. Patient’s prognostic preferences	0.106	0.050	2.325	0.022	
TIOS. Trust in oncologist	0.548	0.195	5.743	0.001	
Age	0.005	0.008	0.287	0.774	
Sex	0.505	0.035	1.291	0.197	
Tumor site	0.101	0.018	0.681	0.496	

Abbreviations: STAR-P, Scale to assess the Therapeutic Relationship-Patients version; CAHPS, Satisfaction with decision-making and physician communication; PPD, Preference of Patients to know Prognosis; TiOS, Trust in the oncologist scale. B: unstandardized coefficients; Beta: standardized coefficients.

## Data Availability

The datasets generated during and analyzed during the current study are not publicly available for reasons of privacy. However, they are available (fully anonymized) from the corresponding author upon reasonable request.

## Appendix A

**Table A1:** List of 15 centers participants in the study.

(1) Hospital Universitario de Canarias, Tenerife (2) Hospital Universitario La Paz, Madrid (3) Hospital Universitario Central de Asturias, Oviedo (4) Complejo Hospitalario Universitario de Ourense (5) Hospital Universitario Infanta Leonor, Madrid (6) Consorcio Hospital General Universitario de Valencia, Valencia (7) Hospital General Virgen de la Luz, Cuenca (8) Hospital Provincial de Castellón, Castellón (9) Hospital General Universitario de Elche, Elche (10) Hospital Universitario Clínico San Carlos, Madrid (11) Hospital San Pedro de Alcántara, Cáceres (12) Hospital Universitario Morales Meseguer, Murcia (13) Hospital Quironsalud Sagrado Corazón de Sevilla, Sevilla (14) Hospital General Universitario Santa Lucia, Cartagena (15) Hospital General de Ciudad Real, Ciudad Real
